# ECMDB 2.0: A richer resource for understanding the biochemistry of *E. coli*

**DOI:** 10.1093/nar/gkv1060

**Published:** 2015-10-19

**Authors:** Tanvir Sajed, Ana Marcu, Miguel Ramirez, Allison Pon, An Chi Guo, Craig Knox, Michael Wilson, Jason R. Grant, Yannick Djoumbou, David S. Wishart

**Affiliations:** 1 Department of Computing Science, University of Alberta, Edmonton, AB, T6G 2E9, Canada; 2 Department of Biological Sciences, University of Alberta, Edmonton, AB, T6G 2E8, Canada; 3 National Institute for Nanotechnology, 11421 Saskatchewan Drive, Edmonton, AB, T6G 2M9, Canada

## Abstract

ECMDB or the *Escherichia coli* Metabolome Database (http://www.ecmdb.ca) is a comprehensive database containing detailed information about the genome and metabolome of *E. coli* (K-12). First released in 2012, the ECMDB has undergone substantial expansion and many modifications over the past 4 years. This manuscript describes the most recent version of ECMDB (ECMDB 2.0). In particular, it provides a comprehensive update of the database that was previously described in the 2013 NAR Database Issue and details many of the additions and improvements made to the ECMDB over that time. Some of the most important or significant enhancements include a 13-fold increase in the number of metabolic pathway diagrams (from 125 to 1650), a 3-fold increase in the number of compounds linked to pathways (from 1058 to 3280), the addition of dozens of operon/metabolite signalling pathways, a 44% increase in the number of compounds in the database (from 2610 to 3760), a 7-fold increase in the number of compounds with NMR or MS spectra (from 412 to 3261) and a massive increase in the number of external links to other *E. coli* or chemical resources. These additions, along with many other enhancements aimed at improving the ease or speed of querying, searching and viewing the data within ECMDB should greatly facilitate the understanding of not only the metabolism of *E. coli*, but also allow the in-depth exploration of its extensive metabolic networks, its many signalling pathways and its essential biochemistry.

## INTRODUCTION

*Escherichia coli* is a rod-shaped, Gram-negative, enteric bacterium that is commonly found in the large intestine or hind-gut of most mammals. It can live both aerobically or anaerobically, making it a very adaptable facultative anaerobe. More than 3000 *E. coli* strains are known and 127 have been fully sequenced to date. While most *E. coli* strains are harmless, some serotypes (such as the serotype O157) can cause severe food poisoning. The most commonly studied and certainly the most commonly used *E. coli* strain is K-12 (MG1655), a harmless, laboratory-derived strain that has proven to be particularly amenable to genetic manipulation (1). Its genome was fully sequenced in 1997 (2) and it consists of a single, circular chromosome with 4.639 million base pairs encoding for nearly 4500 genes (4282 protein genes and 206 RNA genes) in approximately 650 operons (3). *E. coli* (K-12) along with the mouse, rat, zebrafish, fruitfly, nematode, Arabidopsis and baker's yeast, is part of an ‘elite’ set of less than a dozen model organisms that serve as both ubiquitous laboratory ‘tools’ and universal references for the study of nearly all other related classes of organisms. As the only bacterium in this set of model organisms, *E. coli* has long served as a template for understanding or interpreting the biology and biochemistry of nearly all bacteria. As a result, *E. coli* has become the world's most intensively studied prokaryote. The knowledge acquired from more than 60 years of in-depth study of this organism is now contained in a number of excellent *E. coli*-specific resources including online databases such as EcoCyc (4), EchoBase (5), the CyberCell Database (CCDB) (6), EcoGene (7) and RegulonDB (8). The detailed metabolic knowledge derived from *E. coli* has also made its way into a number of other, more general pathway and metabolism databases such as Reactome (9), MetaCyc (10) and the Kyoto Encyclopedia of Genes and Genomes (KEGG) (11).

Unfortunately, as a number of recent metabolomic studies have shown, many of these metabolism and pathway databases are still missing large numbers of *E. coli* metabolites as well as many important metabolite classes (12–15). Furthermore, most existing *E. coli* databases lacked the kind of reference information and database query tools needed by modern metabolomics researchers, such as online MS (Mass Spectrometry) or NMR (Nuclear Magnetic Resonance) spectral searching, detailed metabolite descriptions, lists of metabolite concentrations, substrate growth conditions as well as other kinds of data that are routinely needed to study *E. coli* metabolism or *E. coli* metabolomics. In an effort to address these shortcomings and with a goal to create a database more targeted to the needs of *E. coli* (and bacterial) metabolomics, we developed ECMDB (16). While the first version of ECMDB was very well received by metabolomics researchers, there were still a number of shortcomings to the database that prevented it from appealing to a broader community. In particular, it only had about 50% of its metabolites linked to known metabolic pathways and an even smaller number linked to known signalling pathways or known transporters. Furthermore, only a small fraction (<20%) of ECMDB's compounds had referential NMR or MS spectral data.

By taking advantage of recent developments in machine-readable pathway rendering and viewing (17,18) as well as an enormous increase in the number of tools for spectral prediction (19–21) and spectral deposition (22,23) we have been able to correct these deficiencies. Furthermore, by adding the newly published chemical/metabolomic data about *E. coli* and incorporating many other advances web interface technologies for online databases (24), we have been able to substantially add to both the content and capabilities of ECMDB. This manuscript provides a detailed description of these improvements along with a point-by-point comparison between the original ECMDB and ECMDB 2.0 (the most recent version).

## DATABASE UPDATES AND ENHANCEMENTS

There are five major improvements that are featured in ECMDB 2.0. These include: (i) significantly more and greatly improved pathway diagrams; (ii) increased transport protein, enzyme, reaction and metabolite coverage; (iii) much more reference (MS and NMR) spectral data along with significantly more spectral information and spectral viewing capabilities; (iv) enhanced information about all of ECMDB's chemical compounds (synthesis, safety, taxonomy, ontology, etc.); and (v) significant improvements to the interface (responsiveness, query speed, ease-of-use). Many of these enhancements are outlined in Table 1, which compares the original version of ECMDB with ECMDB 2.0. Additional details for each of the five major improvements are given below.

**Table 1.** Comparison between ECMDB 1.0 and ECMDB 2.0

Category	ECMDB 1.0	ECMDB 2.0
Number of metabolites	2610	3760
Number of reactions	3145	8097
Number of pathways	125	1650
Number of enzymes	1271	1611
Number of transporters	278	706
Number of proteins	1549	2133
Number of metabolite synonyms	25 212	37 982
Number of metabolite references	4440	8591
Number of MSDS sheets	0	380
Number of spectra	4810	22 158
Number of compounds with 1D NMR spectra	103	564
Number of compounds with 2D NMR spectra	338	457
Number of compounds with NMR or MS spectra	412	3261
Number of NMR spectra	775	1487
Number of MS spectra	4035	20 671
Number of predicted MS/MS spectra	0	15 648
Number of pathways from Pathwhiz/SMPDB	0	1225
Number of reactions from Pathwhiz/SMPDB	0	3030
Percentage of metabolites in reactions	35%	90%
Percentage of metabolites in pathways	54%	90%

## PATHWAY IMPROVEMENTS

The original version of ECMDB contained links to 125 *E. coli* KEGG pathways. However, this provided pathway coverage of only 50% out of the 2610 compounds in ECMDB. While the KEGG database is an exceptional resource, it lacks key information about most aspects of lipid metabolism, metabolite localization, metabolite transport and metabolite signalling or regulation (i.e. operon) pathways. Furthermore, the KEGG pathways do not maintain reciprocal links to the metabolite data in ECMDB. It was also notable that the original version of ECMDB did not have links to the ∼300 pathways found in EcoCyc. We have corrected this oversight with ECMDB 2.0, but we have also extended ECMDB's pathway collection much further. In particular, recent advances in JavaScript technology have allowed the creation of rich, interactive user interfaces that can be used to display complex wiring diagrams and maps (i.e. Google Maps). We applied these concepts to create a web-enabled tool called PathWhiz (18) for generating detailed, colorful, interactive and machine-readable (SBML, BioPAX, SBGN-ML, PWML) pathway diagrams. This tool was subsequently used to create a large database of >800 human metabolic pathways for the Small Molecule Pathway Database or SMPDB (17).

Using PathWhiz, we have now generated a total of 1225 interactive *E. coli* pathways including 1203 metabolic pathways and 22 signalling (operon) pathways. Additionally, ECMDB 2.0 now has links to 138 KEGG pathways and 312 EcoCyc pathways. This means that nearly every metabolite (90%) in ECMDB 2.0 is now linked to a pathway and every element in ECMDB's’ pathways is linked to either a compound in ECMDB or a protein in UniProt (25). Each PathWhiz diagram depicts detailed information about the cellular and subcellular locations of the pathway components, the active proteins (hyperlinked to UniProt IDs), the protein quaternary structures (if known), protein cofactors (if present), reaction processes (shown by arrows), the protein positions within the cell (membrane, cytoplasm, periplasm) and the metabolite products or reactants (hyperlinked to ECMDB IDs). Furthermore, every pathway is given a meaningful title along with a detailed, carefully written description of the pathway that is depicted in the diagram. By using PathWhiz, our annotation team was able to generate pathway diagrams that included transport proteins and transport reactions occurring between the outer membrane, periplasmic space and inner membrane of the *E. coli* cell – something not depicted in any pathway database of which we are aware.

**Figure 1.**
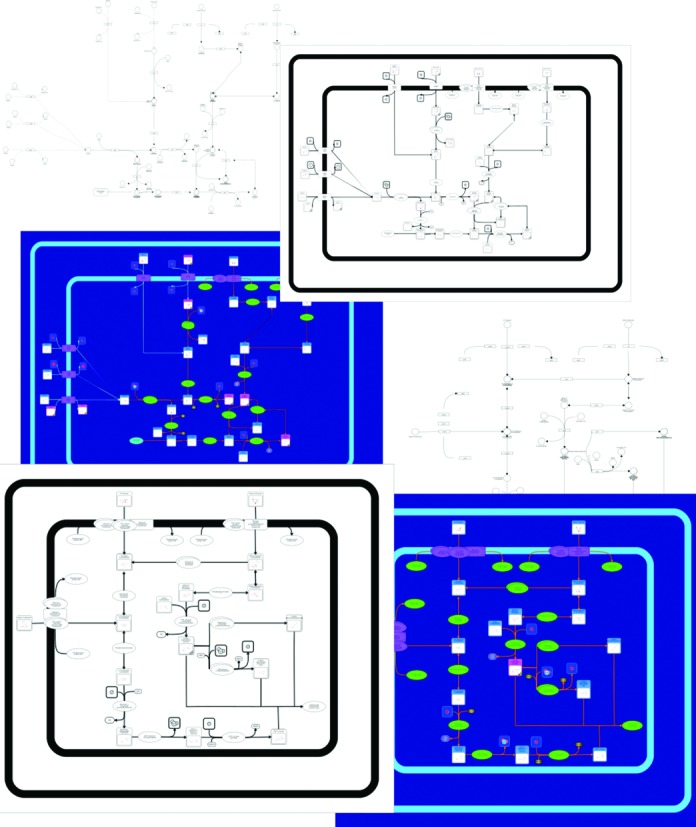
A screenshot montage of the *Escherichia coli* Metabolome Database (ECMDB) showing several of its pathways. The top three pathways correspond to Colanic Acid Biosynthesis pathway and the bottom three pathways refer to Galactose Metabolism pathway.

Each ECMDB pathway is now viewable through the PathWhiz viewer and each pathway can be displayed as either: (i) a richly colored image with explicit metabolite and protein structure depictions; (ii) a black-and-white version; and (iii) a simplified KEGG-like or Reactome-like wiring diagram with proteins depicted as boxes and metabolites depicted as circles. Examples of these pathway image formats are shown in Figure 1. All pathway images may be interactively zoomed or recolored to display metabolite concentration levels. Likewise, all pathways may be downloaded as PNG or higher resolution SVG files for figure placement into papers or slide presentations. By striving to generate as many complete and accurate pathway diagrams as possible, which cover as much of *E. coli* metabolism as possible, we believe we have laid a much more solid foundation for depicting and understanding *E. coli*'s biochemistry. Indeed, this massive pathway generation effort led to the fortuitous discovery of a number of ‘necessary’ intermediate metabolites that had not previously been included in other *E. coli* databases (including ECMDB).

## INCREASED COVERAGE

As noted above, the pathway generation effort for ECMDB 2.0 led to the identification and subsequent addition of hundreds of new metabolites, proteins and reactions into the database. This included many metabolic intermediates for lipid synthesis as well as a large number of proteins and metabolites involved in operon function, metabolite signalling and metabolite transport. In addition, our curation team also spent a great deal of time scanning existing online *E. coli* metabolism databases for recent updates as well as reviewing with the recent *E. coli* literature (from 2011 onward) to identify additional metabolites, enzymes and reactions that were newly described or identified and not already in ECMDB. Altogether, this led to the addition of 1150 new metabolites, 564 new proteins or enzymes and 4952 reaction/transport annotations (Table 1). This represents a 44.1%, 37.7% and 157.5% increase, respectively, over what was originally contained in the first version of ECMDB.

Our decision to include transport proteins, transport reactions as well as many proteins and reactions involved in metabolite signalling in ECMDB 2.0 is indicative of an important paradigm shift regarding metabolites and metabolism within the metabolomics community. Traditionally, metabolites have been viewed simply as the bricks and mortar of cells, which led to a near exclusive focus on their roles in anabolic and catabolic reactions (as depicted in most biochemistry textbooks). More recently, metabolites are being recognized as vital switches and key signalling molecules that play an important role in directing or controlling many cell functions, including transcription, translation, cell detection, cell movement and many cell-fate decisions. This expanded view of metabolites and their biological roles will no doubt lead to the identification and generation of many more pathways, reactions and components that will have to be added to the next release of ECMDB.

## ENHANCED SPECTRAL INFORMATION

In metabolomics, the identification of metabolites is critically dependent on the availability of reference NMR, MS/MS or GC-MS spectral data of pure compounds. As a result, most metabolomics databases such as ECMDB, HMDB (the Human Metabolome Database) or Metlin (16,26,27) place a high premium on providing up-to-date reference spectral data that can be easily searched, queried or viewed. The original version of ECMDB contained a total of 4810 experimentally acquired NMR spectra and MS spectra from a total of 412 compounds. With the deployment of many new open-access spectral database resources such as the BioMagResBank (23) and MassBank (22) as well as significant improvements to the ability to predict MS/MS spectra with programs such as MetFrag (21) and CFM-ID (19), we decided to take this opportunity to significantly update and upgrade the spectral data in ECMDB. The current version of ECMDB now contains 712 additional experimental NMR spectra, 988 additional experimental MS, MS/MS and GC/MS spectra and 15,648 predicted (via CFM-ID) MS/MS spectra (Table 1). For the NMR and MS spectra, this represents a 91.9% and a 412.3% increase over what was originally contained in the original version of ECMDB. It is also worth noting that all 3760 metabolites in ECMDB 2.0 now have either experimental or predicted MS/MS spectra.

In addition to the increased quantity of spectral data within ECMDB 2.0, we have also made significant improvements to the quality and viewability of its reference spectral images. In the original version of ECMDB, all images of the experimentally acquired NMR spectra were static PNG files. ECMDB 2.0 now displays all of its NMR spectra in an interactive JavaScript spectral viewer called JSpectraViewer. With this viewer, all NMR spectra can be interactively zoomed, rescaled or otherwise manipulated with a simple click of a button. In a similar vein, all of the MS/MS and EI-MS spectra in ECMDB 2.0 have been recently upgraded and regenerated so that they may be viewed as interactive ‘stick’ images. A number of these updated MS/MS images are also annotated with fragment ion structures so that as the user mouses over the peaks, the predicted fragment ions appear.

## RICHER DATA CONTENT

Our curation team has continued to develop a number of in-house tools that automatically gather, annotate, compile, generate, predict (chemical properties), correct or classify chemical compounds based on their names, InChI (International Chemical Identifier) code or structure. Using an in-house tool called DataWrangler, we validated, re-annotated and corrected every compound in ECMDB 2.0. Starting with a compound name, CAS (Chemical Abstract Service) number or InChI identifier DataWrangler searches through more than a dozen online chemical data resources and performs a variety of predictions, annotations and ‘sanity checks’ on each query before compiling a summary of its findings, corrections, predictions and suspected inconsistencies. In this way, all of ECMDB's structures, chemical names, synonyms, predicted chemical properties, database links and related data were updated, more fully annotated and/or corrected. Additionally, DataWrangler also captures synonyms, external links, chemical synthesis references and additional PubMed references regarding the compound. As a result, ECMDB 2.0 now has 12 720 more synonyms, 2085 more external links for compounds and 4151 more references than the previous version.

Moreover, using ClassyFire, a tool for structure-based taxonomic classification of chemicals, we classified every compound in ECMDB 2.0. Each chemical class in the taxonomy has a detailed text description (an ontology), which emphasizes the main structural characteristics of the compounds it contains. In addition to the taxonomic classification by ClassyFire, all chemicals in ECMDB 2.0 were also assigned their respective classification (when available) as provided by ChEBI (28), KEGG (11), MetaCyc (10), or LIPID MAPS (29). The use of a standardized chemical taxonomy/ontology is very useful for comparing, clustering or describing groups of metabolites. Chemical taxonomies are also being used in metabolite set enrichment tools, and in a variety of machine learning applications in cheminformatics.

## IMPROVED USER INTERFACE

In addition to the substantial expansion and improvement of ECMDB's content, we have also made a number of improvements to ECMDB's user interface. These changes have substantially enhanced the database's responsiveness, query speed and ease-of-use. For instance, code optimization has accelerated the NMR and MS/MS spectral search speeds by up to 500X over the original version. The use of JavaScript tools to display NMR spectra, MS spectra and pathway image data has also made for a much more colorful, informative and interactive viewing experience. ECMDB's text searches have been modified to support Elasticsearch. Elasticsearch allows for ‘approximate’ text queries where typos and mis-spellings are both tolerated and properly reinterpreted.

ECMDB 2.0 also takes advantages of a number of recent improvements to web-based tools, frameworks and caching systems to make the website more user friendly and responsive. The design is inspired by the Twitter Bootstrap framework and it makes for much easier navigation and a more appealing user experience. The server uses Redis-based caching that makes the loading of compounds, protein reactions and pathways very fast. In particular, the wait times for flushing a compound annotation page or a protein annotation page has been reduced by a factor of four or more. Each ECMDB compound view page now includes a jump-tab (the row of buttons on top of a compound annotation page) that allows users to quickly jump to specific data fields. In addition to these jump-tabs, we have also added filters to the ‘Browse Compounds’ page. These filters allow for the rapid selection of a more specialized list of compounds according to the user's needs.

To facilitate rapid prototyping and development, the entire ECMDB database has been built upon an MVC (Model-View-Controller) framework called Ruby on Rails. In the MVC framework, models respond and interact with the data by connecting to the database, views create the interface to show and interact with the data, and controllers connect the user to the views. Such a framework allowed our programmers to easily create code for each of the respective modules in ECMDB. The design is particularly robust and code can be reused in different functions or changed easily to accommodate future plans or abrupt changes in design. In particular, this allowed our development team to liberally borrow code and functions from T3DB's (24) recently enhanced interface.

In addition to the many enhancements to the user interface, we also improved ECMDB's data accessibility. Each compound annotation page has an ‘XML’ button that when clicked will display all the compound's annotations in XML format. Individual pages or the entire ECMDB database may be downloaded as an XML-formatted file. For those who prefer a simpler or less verbose data format, all of the data in ECMDB 2.0 can also be viewed or downloaded in the more compact ‘JSON’ format. To further facilitate downloading, the entire ECMDB database is now downloadable via ‘HTTP Get’ access. This allows users to easily download all of the ECMDB data including concentration information, chemical classification data, synonyms, spectral data and structural data.

## CONCLUSION

The ECMDB version 2.0 represents a substantial enhancement to the original version of ECMDB. Perhaps the most significant improvement is the 12-fold increase in the number of metabolic pathway diagrams (from 125 to 1650) now housed in the database. These pathway diagrams not only cover almost all aspects of *E. coli* metabolism but they also capture many aspects of *E. coli's* metabolite signalling and metabolite transport activities. Compared to other resources such as KEGG (with 138 *E. coli*-specific pathways) or EcoCyc (with 337 *E. coli*-specific pathways), ECMDB 2.0 with its 1650 metabolic and signalling pathways now provides the most complete and comprehensive pathway description of any known organism.

Many other improvements or enhancements were also implemented into ECMDB 2.0. These include a 3-fold increase in the number of compounds linked to pathways (from 1058 to 3280), a 44% increase in the number of compounds in the database (from 2610 to 3760), a significant increase in the number of NMR and MS spectra (from 4810 to 22 158) and a massive increase in the number of types of external links to other *E. coli* or chemical resources. These additions, along with many user interface enhancements aimed at improving the ease or speed of querying, searching and viewing ECMDB's data are expected to greatly facilitate *E. coli* (or other bacterial) metabolomic studies. They are also expected to encourage a far more in-depth exploration and understanding of *E. coli's* extensive metabolic networks, its many signalling pathways and its essential biochemistry.

By concentrating on filling many of the ‘holes’ in the original ECMDB annotations and pathways we have been able to establish a fairly complete and comprehensive ‘machine-readable’ picture of bacterial biochemistry. By using this information as a template, we plan to map the metabolomic, genomic, proteomic and pathway information contained in ECMDB to a large number of common enteric (gut) bacteria. This multi-year effort (called ‘microbes to metabolites’ or M2M) will ultimately lead to the comprehensive automated or semi-automated annotation of more than 500 bacteria associated with the human gut microbiome.
